# Roles of histone deacetylases in epigenetic regulation: emerging paradigms from studies with inhibitors

**DOI:** 10.1186/1868-7083-4-5

**Published:** 2012-03-12

**Authors:** Geneviève P Delcuve, Dilshad H Khan, James R Davie

**Affiliations:** 1 Manitoba Institute of Cell Biology, University of Manitoba, 675 McDermot Avenue, Winnipeg, MB, R3E 0V9, Canada

**Keywords:** histone deacetylase, HDAC, HDAC inhibitors, HDAC complexes, gene expression, noncoding RNAs, epigenetics

## Abstract

The zinc-dependent mammalian histone deacetylase (HDAC) family comprises 11 enzymes, which have specific and critical functions in development and tissue homeostasis. Mounting evidence points to a link between misregulated HDAC activity and many oncologic and nononcologic diseases. Thus the development of HDAC inhibitors for therapeutic treatment garners a lot of interest from academic researchers and biotechnology entrepreneurs. Numerous studies of HDAC inhibitor specificities and molecular mechanisms of action are ongoing. In one of these studies, mass spectrometry was used to characterize the affinities and selectivities of HDAC inhibitors toward native HDAC multiprotein complexes in cell extracts. Such a novel approach reproduces *in vivo *molecular interactions more accurately than standard studies using purified proteins or protein domains as targets and could be very useful in the isolation of inhibitors with superior clinical efficacy and decreased toxicity compared to the ones presently tested or approved. HDAC inhibitor induced-transcriptional reprogramming, believed to contribute largely to their therapeutic benefits, is achieved through various and complex mechanisms not fully understood, including histone deacetylation, transcription factor or regulator (including HDAC1) deacetylation followed by chromatin remodeling and positive or negative outcome regarding transcription initiation. Although only a very low percentage of protein-coding genes are affected by the action of HDAC inhibitors, about 40% of noncoding microRNAs are upregulated or downregulated. Moreover, a whole new world of long noncoding RNAs is emerging, revealing a new class of potential targets for HDAC inhibition. HDAC inhibitors might also regulate transcription elongation and have been shown to impinge on alternative splicing.

## Introduction

Acetylation of the lysine ε-amino group, first discovered on histones, is a dynamic posttranslational modification (PTM) regulated by the opposing activities of lysine acetyltransferases (KATs) and histone deacetylases (HDACs). Histone acetylation is a modulator of chromatin structure involved in DNA replication, DNA repair, heterochromatin silencing and gene transcription [1,2]. Hyperacetylation usually marks transcriptionally active genes, as it contributes to the decondensed chromatin state and maintains the unfolded structure of the transcribed nucleosome [2-6]. Moreover, specific acetylated sites on core histones are read by bromodomain modules found in proteins, and sometimes in KATs, which are components of chromatin-remodeling complexes involved in transcriptional activation [7]. Conversely, HDACs are found in corepressor complexes and, by removing acetyl groups from histones, induce the formation of a compacted, transcriptionally repressed chromatin structure. As discussed below, however, this model reflects quite an oversimplification of the role of HDACs in transcription regulation.

Many nonhistone proteins (transcription factors, regulators of DNA repair, recombination and replication, chaperones, viral proteins and others) are also subject to acetylation [8-10]. Investigators in a recent study used high-resolution mass spectrometry to identify 3,600 acetylation sites in 1,750 human proteins and showed that lysine acetylation is implicated in the regulation of nearly all nuclear functions and many cytoplasmic processes [11]. Furthermore, acetylation is regulated by and/or regulates other PTMs. Through either recruitment or occlusion of binding proteins, PTMs may lead to or prevent a secondary PTM on histones and nonhistone proteins [12,13]. In particular, histone H3 phosphorylation on serine 10 or 28, rapid and transient PTMs in response to the stimulation of signaling pathways such as the mitogen-activated protein kinase (MAPK) pathways, are associated with histone acetylation and transcriptional activation of specific genes [14]. A cross-talk also exists between histone acetylation and H3 methylation. Although acetylation is generally linked to transcription activation, the effect of methylation depends on which amino acid residue is modified and the degree to which this residue is methylated (mono-, di- or trimethylation of lysine). Methylation of H3 lysine 4 or 36 is associated with transcription activation, but methylation of lysine 9 or 27 is linked to transcription repression [15,16].

To date, 18 different mammalian HDACs have been identified and divided into four classes based on their sequence similarity to yeast counterparts [17,18]. HDACs from the classical family are dependent on Zn^2+ ^for deacetylase activity and constitute classes I, II and IV. Class I HDACs, closely related to yeast RPD3, comprise HDAC1, HDAC2, HDAC3 and HDAC8. Class II HDACs, related to yeast HDA1, are divided into subclass IIa (HDAC4, HDAC5, HDAC7 and HDAC9) and subclass IIb (HDAC6 and HDAC10). Class IV contains only HDAC11. Class III HDACs consist of seven sirtuins, which require the *NAD^+ ^*cofactor for activity.

Inhibitors of Zn^2+^-dependent HDACs were originally discovered as inducers of transformed cell growth arrest and cell death and only later were identified as inhibitors of HDAC activity [19]. It was recognized that HDACs are upregulated in many cancers or aberrantly recruited to DNA following chromosomal translocations, particularly in hematologic malignancies [20,21]. The specificity of HDAC inhibitors toward tumor cells, although poorly understood, has led to their development as anticancer drugs. More recently, clinical studies using HDAC inhibitors have been extended to a range of nononcologic diseases, such as sickle cell anemia, HIV infection, cystic fibrosis, muscular dystrophy and neurodegenerative and inflammatory disorders [21-23]. The use of HDAC inhibitors also constitutes a chemical approach to the study of HDAC cellular functions. In addition, crucial developmental and physiological roles of HDACs have been elucidated by knockout studies [21,24]. The scope of this review concerns emerging concepts regarding the roles of HDACs in modulating chromatin structure and function as revealed by studies with HDAC inhibitors.

### Class I HDAC complexes

Class I HDACs are ubiquitously expressed nuclear enzymes, although HDAC8 is generally poorly expressed [17]. Except for HDAC8, class I HDACs are components of multiprotein complexes. Knockout studies have shown that class I HDACs are involved in cell proliferation and survival [21,24]. As products of an evolutionary recent gene duplication, HDAC1 and HDAC2 exhibit a high degree of homology (85% identity for human proteins) [18,25] and have undergone little functional divergence, although specific and distinct roles have also been identified for each of them [26,27]. For example, targeted disruption of the *Hdac1 *alleles in mouse results in cell proliferation defects and embryonic lethality by embryonic day E9.5 [28], whereas mice lacking HDAC2 survive at least until the perinatal period [29-31]. On the basis of their differential distributions in the brain at distinct stages of neuroglial development, HDAC1 and HDAC2 appear to have different functions during the development of the central nervous system [32]. Moreover, HDAC2, but not HDAC1, negatively regulates memory formation and synaptic plasticity [33]. Surprisingly, researchers in a recent study suggested that HDAC1 has a protective role against the formation of immature teratomas with high malignant potential in both mouse studies and human patients [34].

HDAC1 and HDAC2 form homo- and heterodimers between each other [26,35,36], which presumably allows them to act together or separately from each other. The dimer is a requirement for HDAC activity [36]. Dissociation of the dimer with a HDAC1 N-terminal peptide will inhibit HDAC activity [36]. Viruses have capitalized on this mechanism to inhibit HDAC activity. The adenoviral protein GAM1 inhibits HDAC1 activity by binding to the N-terminal region of HDAC1, which likely dissociates the dimer [37].

HDAC1 and HDAC2 heterodimer levels seem to depend on the cell type, because it was shown that 80% to 90% of HDAC1 and HDAC2 proteins were associated with each other in the nucleus of human breast cancer MCF-7 cells [38], whereas 40% to 60% of HDAC1 and HDAC2 proteins were found to be free from each other in mouse embryonic fibroblasts [39]. Moreover, a genomewide mapping study in primary human CD4^+ ^T cells revealed a differential distribution of HDAC1 and HDAC2 along regulatory and coding regions [40]. Conversely, HDAC1 and HDAC2 were both associated with regulatory and coding regions in MCF-7 cells [38,41]. HDAC1 and HDAC2 relative expression levels also vary with cell types. For example, T-lymphocyte Jurkat cells express negligible levels of HDAC2 compared to HDAC1 levels [42], and throughout the adult brain HDAC2 is preferentially expressed in neurons, whereas HDAC1 is more abundant in glial cells [32,33]. It is likely that, at least in cells expressing markedly different relative levels of HDAC1 and HDAC2, homodimer formation would prevail over heterodimer formation.

**Figure 1:**
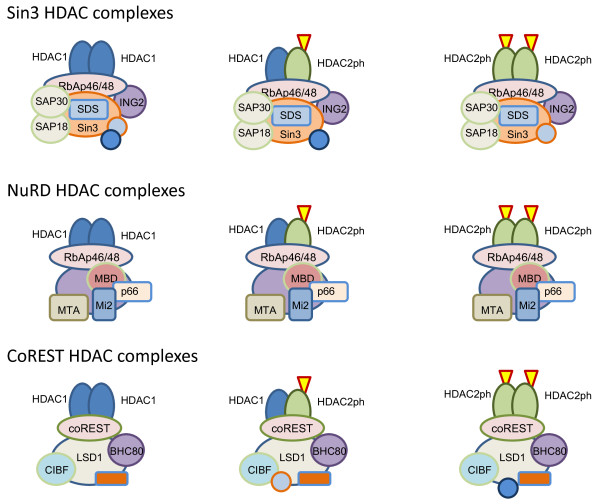
**Class I HDAC1-HDAC2 multiprotein complexes**. Multiprotein complexes containing HDAC1-HDAC2 homo- or heterodimers are shown. HDAC2 is shown as phosphorylated, which is a requirement for multiprotein complex formation. Phosphorylation is indicated by a red-outlined yellow triangle.

HDAC1 and HDAC2 are both found in multiprotein corepressor complexes Sin3, nucleosome-remodeling HDAC (NuRD) and CoREST, which are recruited to chromatin regulatory regions by transcription factors (for example, Sp1, Sp3, p53, NF-κB and YY1) and have very diverse, often cell-specific, roles (Figure 1) [17,43]. Although it is generally assumed that both HDACs can be paired within the same complex, to the best of our knowledge, it has been demonstrated only in studies using exogenously expressed, tagged HDAC1 and not in studies characterizing endogenous HDAC corepressor complexes [36]. The Sin3 core complex contains Sin3A or Sin3B, HDAC1 and/or HDAC2, SAP18, SAP30 and retinoblastoma-associated proteins (RbAps) RbAp46 and RbAp48 and serves as a platform for the addition of other modules with enzymatic functions such as nucleosome remodeling, DNA methylation, histone methylation and N-acetylglucosamine transferase activity [44,45].

The NuRD complex has a variable composition that is dependent on the cell type and external stimuli. It is the only complex holding both HDAC- and ATP-dependent chromatin-remodeling activities, which are carried out by HDAC1 and/or HDAC2 and Mi-2α and/or Mi-2β, respectively. The other known components of NuRD are structural and/or regulatory proteins RbAp46/RbAp48 and, in some instances, also p66α or p66β, the methyl-CpG-binding domain-containing proteins (MBD2 or MBD3), with only MBD2 being able to recognize methylated DNA and the three members of the metastasis-associated protein family (MTA1, MTA2 or MTA3), with different MTA proteins allowing distinct downstream responses to the activation of different signaling pathways [45,46]. Lysine-specific demethylase 1 (KDM1/LSD1) has also been identified as a component of NuRD [47].

HDAC1 and HDAC2 are also components of the Nanog- and Oct4-associated deacetylase (NODE) complex, a NuRD-related repression complex, also comprising MTA1 or MTA2, p66α or p66β, but not the histone-binding proteins RbAp46/RbAp48 and the helicase-like ATPase Mi-2. NODE is involved in the control of embryonic stem cell fate by repressing Nanog and Oct4 target genes [48].

Also including HDAC1 and HDAC2, but composed of proteins distinct from those of Sin3 and NuRD, the CoREST complex is recruited by the RE1 silencing transcription (REST) factor, also known as the "neuronal restricted silencing factor" (NRSF), to the RE1 DNA motif associated with many genes encoding fundamental neuronal traits. As a component of the CoREST complex and as a consequence of histone H3 deacetylation, KDM1/LSD1 promotes demethylation of H3 dimethylated on lysine 4 (H3K4me2), an event that facilitates the formation of a repressive chromatin structure [49,50]. Although CoREST acts as a corepressor in terminally differentiating nonneuronal cells by recruiting KDM1/LSD1 to demethylate H3K4me2 and the methyltransferase G9a to methylate H3K9 at the RE1 sites of target genes, it acts as a coactivator of transcription in embryonic stem cells and neural stem cells by recruiting an H3K4 methyltransferase to the RE1 sites of target genes [51]. CoREST can also form larger complexes by association with ZNF217, a Krüppel-like zinc finger protein and strong candidate oncogene product found in breast cancer, or with other complexes, such as the chromatin-remodeling complex SWI/SNF or the C-terminal binding protein (CtBP) complex [45,52]. Interestingly, CoREST appears to be involved in the negative regulation of synaptic plasticity and memory formation by HDAC2 [33]. Chromatin immunoprecipitation (ChIP) and immunocoprecipitation experiments performed on mouse forebrains showed that, though both HDAC1 and HDAC2 were incorporated in Sin3 and NuRD complexes and were found to be enriched at the promoters of cell-cycle genes, HDAC2 was preferentially associated with the CoREST complex to repress neuronal gene expression [33].

A novel HDAC complex, MiDAC, is specific to mitotic cells and includes HDAC1, HDAC2, either one of the related ELM-SANT proteins MIDEAS or TRERF1, and DNTTIP1 (terminal deoxynucleotidyl transferase (TdT)-interacting protein), although the authors who published these findings suggested that the MiDAC complex has a TdT-independent function in cell division. Whether the putative histone acetylase CDYL is also a MiDAC component is presently unclear [53].

The discussion above illustrates that the HDAC1 and HDAC2 homo- or heterodimer can exist with different proteins. The combination of these proteins likely determines the overall activity, substrate specificity and genomic location of the HDAC1 and/or HDAC2 containing complex.

The two highly related complexes nuclear receptor corepressor (NCoR or NCOR1) and silencing mediator of retinoic acid and thyroid hormone receptor (SMRT or NCOR2) consist of HDAC3, transducin β-like 1 (TBL1), TBL-related 1 (TBLR1) and G protein pathway suppressor 2 (GPS2) [45,54]. NCoR and SMRT also interact with class IIa HDACs, which exhibit no deacetylase activity of their own but are believed to recruit NCoR/SMRT HDAC3 activity to distinct promoters through their associated factors, such as myocyte enhancer factor 2 (MEF2) [55]. NCoR, but not SMRT, interacts with zinc finger and BTB domain-containing 33 (ZBTB33 or Kaiso), which is a protein that binds to methylated DNA. NCoR and SMRT are regulated by different kinase pathways and play different roles in development. Although NCoR binds preferentially to the thyroid hormone receptor, SMRT prefers the retinoic acid receptor [45,54]. It is noteworthy that repression by NCoR/SMRT is an integral phase of the cyclical process that is the transcriptional activation of genes controlled by liganded receptors. NCoR/SMRT repression is necessary to prime chromatin for subsequent transcription initiation [56]. TBL1 and TBLR1 are involved in the active dismissal of corepressor complexes [54]. Besides its role in transcriptional control, the HDAC3-NCoR-SMRT axis is critical to the maintenance of heterochromatin content and genomic stability [57].

Histone deacetylation also acts in concert with the Polycomb repressive complexes (PRC1/PRC2) or the G9a complex, which catalyze the trimethylation of H3K27 or H3K9, respectively. H3K27me3 is a repressive mark that can easily be reverted, thus conferring plasticity to the chromatin structure. The DNA of Polycomb target genes is generally unmethylated, but some genes can undergo *de novo *DNA methylation under certain circumstances, notably in cancer cells. H3K9me3, on the other hand, is associated with DNA methylation and is a stable mark, denoting permanent silencing [58,59]. As mentioned above, the cooperation of DNA methylation and histone deacetylation in gene silencing is also established by the recruitment of complexes such as Sin3 or NuRD via proteins that bind methylated DNA such as MeCP2 or MBD2.

### Class II histone deacetylases

Class II HDACs shuttle between nucleus and cytoplasm and have tissue-specific expression and functions [21,24,43,60]. Class IIa HDACs (HDAC4, HDAC5, HDAC7 and HDAC9) are signal transducers characterized by the presence in their regulatory N-terminal domains of two or three conserved serine residues subject to reversible phosphorylation. Phosphorylation leads to the binding of the 14-3-3 proteins, the nuclear export of HDACs and the derepression of their target genes. A range of kinases and phosphatases acting downstream of diverse biological pathways have been shown to regulate the nucleocytoplasmic trafficking of class IIa HDACs [61]. Because of a substitution of Tyr with His in their catalytic site, class IIa HDACs have negligible intrinsic deacetylase activity but are able to bind acetylated lysine. It has been suggested that, under some circumstances, class IIa HDACs may act as bromodomains, recognizing acetylated lysine in a sequence-dependent context and recruiting chromatin-modifying enzymes to regulate transcription [62]. As mentioned above, class IIa HDAC association with MEF2 provides additional targeting for the SMRT-NCoR complex [55]. Class IIa HDACs also interact with numerous other transcription factors. However, the biological relevance of these associations has been established only for the MEF2-regulated processes [63,64]. Class IIa HDACs are not affected by most HDAC inhibitors at pharmacologically relevant concentrations [62].

Class IIb HDACs (HDAC6 and HDAC10) have duplicated catalytic domains, albeit the duplication is partial in the case of HDAC10. HDAC6 and HDAC10 shuttle between nucleus and cytoplasm, but their location is primarily cytoplasmic. Little is known of the role of HDAC10. HDAC6 is an α-tubulin deacetylase as well as a cortactin deacetylase and thus is involved in the control of microtubule- and actin-dependent cell motility. Chaperone protein HSP90 is another substrate of HDAC6. Moreover, HDAC6 plays a critical role in the cellular clearance of misfolded proteins via formation of aggresomes or autophagy [43]. Thus HDAC6 is a potential therapeutic target for the treatment of an array of diseases, including neurodegenerative diseases and cancer [65-67].

### Class IV histone deacetylase 11

HDAC11 has sequence similarity to classes I and II HDACs. Aside from its evolutionary conservation's implying a vital role across species [43] and a study suggesting a role in the decision between immune activation and immune tolerance [68], little is known of HDAC11 functions.

### Selectivity of histone deacetylase inhibitors

The active site of Zn^2+^-dependent HDACs consists of a tubular pocket with two adjacent histidine residues, two aspartic acid residues, one tyrosine residue (substituted with histidine in class IIa HDACs, as mentioned above) and a Zn^2+ ^ion at the bottom of the pocket, all forming a charge-relay system [69]. The HDAC inhibitors currently used in clinical trials or approved by the US Food and Drug Administration fit into this active site pocket, owing to a pharmacophore featuring a Zn^2+^-chelating group and a linker spanning the length of the tubular pocket and connected to a cap that blocks the active site by interacting with the external surface of HDACs. Depending on their chemical Zn^2+^-binding group, HDAC inhibitors belong to different classes, including hydroxamic acids, carboxylic acids, benzamides and cyclic tetrapeptides [19]. A central theme in the literature on HDAC inhibitors is their isoform selectivity or, rather, their perceived lack of isoform selectivity. HDAC inhibitors have generally been considered pan-inhibitors, inhibiting all HDACs from the classical family or class I-specific inhibitors. This view has recently been dispelled, however, by a study revealing no targeting of class IIa HDACs by most HDAC inhibitors [62]. Although it is not known which HDAC isoform's inhibition is responsible for the therapeutic or toxic effects observed in clinical trials, it has generally been assumed that the development of isoform-selective inhibitors would result in preferable clinical outcomes. This theory is unproven to date [20,23]. However, researchers who have performed conventional assays have analyzed the affinities of HDAC inhibitors for different HDACs by using purified HDACs, whereas HDAC activity is mostly associated with multiprotein complexes, the role and composition of which are often cell type-specific. This fact was taken into consideration in a pioneering study in which the investigators carried out the chemoproteomic profiling of 16 HDAC inhibitors with different chemical structures across six human cell lines and six mouse tissues [53]. In that study, a nonselective HDAC inhibitor bound to sepharose beads was added to cell lysates under conditions that preserved the integrity of protein complexes. In a competition assay, the mixture was spiked with a range of concentrations of a free inhibitor interfering with the capture of HDAC complexes by the immobilized inhibitor. Captured proteins were analyzed by quantitative mass spectrometry, and target complexes were reconstituted by matching half-maximal inhibitory concentration values. This initial complex identification was further confirmed by quantitative immunoprecipitation experiments. Although the results collected in this study confirmed that class IIa HDACs were not targeted by any of the studied inhibitors, they mostly conflicted with the isoform selectivity data previously obtained in assays using purified HDACs [62,70]. This is not surprising, in view of a previous kinetic study suggesting that the *in vitro *mode of action of the HDAC inhibitor trichostatin A (TSA) depended on whether the assay conditions preserved HDAC complexes or resulted in their dissociation [71]. Incidentally, it was also shown that TSA did not disrupt HDAC1 and HDAC2 interaction with Sin3A [71]. However, it was shown that TSA and suberoylanilide hydroxamic acid (SAHA, also known as vorinostat), but not less bulky inhibitors such as sodium butyrate or valproic acid, dissociated inhibitor of growth 2 (ING2) from the Sin3 complex, thus disrupting the ING2-mediated recruitment of Sin3 to chromatin [72]. Bantscheff *et al. *[53] found that some inhibitors had different affinities for different complexes. In particular, inhibitors from the benzamide class displayed a higher affinity for the HDAC3-NCoR complex than for NuRD and CoREST complexes, whereas they did not target the Sin3 complex. The affinity of valproic acid, an inhibitor from the carboxylic acid class with moderate potency for class I HDACs, was highest for the CoREST complex, decreased gradually for the NuRD and NCoR complexes and was lowest for the Sin3 complex [53]. The different affinities of HDAC inhibitors detected for different complexes are in agreement with the previous observation that proteins in close proximity to the HDAC active site could interact with the cap of HDAC inhibitors, leading to the suggestion that HDAC-associated proteins could specify inhibitor selectivity [73]. The class IIb enzymes HDAC6 and HDAC10 were inhibited only by hydroxamate compounds. The anti-inflammatory drug bufexamac was identified as a class IIb HDAC inhibitor (HDAC6 and HDAC10), however, and accordingly promoted tubulin hyperacetylation at pharmacologically relevant concentrations. It was shown that hyperacetylation of different HDAC substrates corresponded to the HDAC inhibitor selectivity. For example, class I-selective HDAC inhibitors induced histone but not tubulin hyperacetylation, whereas nonselective HDAC inhibitors stimulated both histone and tubulin hyperacetylation. The authors also identified a novel mitotic HDAC complex, which they called MiDAC [53]. This study provides a new and promising path toward understanding the mode of action of class I-specific HDAC inhibitors and to develop isoform-selective compounds. For example, the combination of this methodology with genomic profiling of the different complexes by ChIP assay (specifically large-scale variants ChIP-on-chip and ChIP-seq) would be highly informative.

### Transcriptional reprogramming by histone deacetylase inhibitors

Inhibition of HDAC activity results in transcriptional reprogramming, which is believed to contribute largely to the therapeutic benefits of HDAC inhibitors on cancers, cardiovascular diseases, neurodegenerative disorders and pulmonary diseases [24]. Inhibition of HDAC enzymatic activity affects the expression of only 5% to 20% of genes, however, with equal numbers of genes being upregulated and downregulated [19]. Only a fraction of these changes are direct effects of HDAC inhibitors, and others are downstream effects, necessitating new protein synthesis. Only some of the direct effects can be inferred as direct consequences of inhibition of histone deacetylation, and others are the results of other mechanisms, such as the inhibition of transcription factor deacetylation, resulting in an altered affinity for DNA binding sites on target gene regulatory regions, an altered interaction with other factors or an altered half-life [74]. Gene expression changes and biological functions targeted by HDAC inhibitors have been thoroughly addressed in recent comprehensive reviews [21,75]. Thus only a few examples of HDAC inhibitor-induced transcriptional changes involving novel or unexpected mechanisms are presented below.

*p21 *(*CDKN1A*), which encodes the cyclin-dependent kinase inhibitor p21, mediating cell cycle arrest, differentiation or apoptosis, is a model gene. Its transcription is directly upregulated in different cell types by different HDAC inhibitors, thus contributing to the antitumor effect of HDAC inhibitors. In parallel with transcriptional activation, a reorganization of chromatin, including histone hyperacetylation, takes place in both the proximal and distal promoter regions [76]. *p21 *is regulated by a variety of factors, including p53. HDAC inhibitor-mediated transcriptional activation is independent of p53, however, and consequently can occur in tumor cells lacking a functional p53. Researchers in a recent study demonstrated that the nucleosomal response to the stimulation of the MAPK signaling pathway was required for *p21 *induction by the HDAC inhibitor TSA. As part of the nucleosomal response, histone H3 in the *p21 *proximal promoter region was phosphorylated on serine 10 by the mitogen- and stress-activated protein kinase 1 (MSK1). It was shown that this phosphorylation event was crucial to the acetylation of neighboring lysine 14. The phosphoacetylation mark was recognized by the 14-3-3ζ protein, reader of phosphoserine marks, and was thus protected from removal by PP2A phosphatase [77]. Presumably, 14-3-3 also acts as a scaffold for the recruitment of chromatin remodeler, leading to initiation of transcription [78]. Additionally, treatment with the HDAC inhibitor depsipeptide, also known as romidepsin, can induce *p21 *expression by causing acetylation of p53, protecting it from ubiquitination-induced degradation and allowing the recruitment of the p300 KAT to the p53-responsive *p21 *promoter [79]. The *p21 *gene may generate several alternate variants [80,81]. The impact of HDAC inhibitors on the genesis of these variants remains to be determined. Some HDAC inhibitors alter pre-mRNA splicing by changing the expression of splicing factors, which are components of the spliceosome. As an example, butyrate, but not TSA, increases the expression of SFRS2 [82]. SFRS2 is required for the expression of *p21 *[82].

The induction of the *Fos *and *Jun *immediate-early genes following the activation of the MAPK pathway is also dependent on MSK-mediated phosphorylation of histone H3 in the promoter region. However, the outcome of HDAC inhibition by TSA on these genes was opposite to the one on *p21 *and opposite to the common belief that histone hyperacetylation is linked to transcription activation. Treatment with TSA resulted in rapid enhancement of H3 acetylation at the promoter region of these genes, but transcription was inhibited [83]. Furthermore, it was shown that continuous dynamic turnover of acetylation was characteristic of genes carrying the active methylation mark on H3K4, but not of genes carrying the repressive methylation mark on H3K9. The authors concluded that acetylation turnover rather than stably enhanced acetylation was crucial to the induction of the *Fos *and *Jun *genes [83]. A similar cyclical process that entails alternating activating and repressive epigenetic events during the hormone-dependent activation of genes has been described [56]. Nonetheless, other scenarios are possible; for example, the transcription activation of *Fos *and *Jun *could require the deacetylation of a nonhistone protein associated with their regulatory region. Investigators in several studies have suggested a role for deacetylation of transcription factors or other proteins in gene induction [84,85]. A proposal for the role of HDACs in the basal transcription from the mouse mammary tumor virus (MMTV) promoter and some other TATA/Inr-containing core promoters is that deacetylation of protein components of the reinitiation complex would allow the recruitment of RNA polymerase II [86].

In another example contrasting with the predominant view of HDACs' being transcriptional repressors, it was shown that HDAC1 served as a coactivator for the glucocorticoid receptor (GR) and that this function was dynamically modulated by acetylation of HDAC1 itself [87]. Researchers in a more recent study reported that HDAC2 was also required for GR-mediated transcriptional activation, and mechanistic insight was presented regarding collaborative regulation by HDAC1 and HDAC2 [36]. HDAC1 and HDAC2 act synergistically in the GR-mediated transcriptional activation at the MMTV promoter. So far, neither their acetylated target nor the nature of the coactivator complex with which they associate has been identified. It was shown that the coactivation function of HDAC1 and HDAC2 is dynamically regulated by acetylation of the HDAC1 C-terminal tail, with K432 being an important site among the six lysine residues that may be acetylated. It is of interest to note that the C-terminal domain of HDAC1 and HDAC2 is a region carrying modifications (phosphorylation and acetylation) that regulate HDAC activity. The acetylation event represses the deacetylase activity of both the HDAC1 homodimer and the HDAC1/HDAC2 heterodimer, even though only HDAC1 is acetylated [36,87]. It was suggested that HDAC1 and HDAC2 form homo- and heterodimers with the catalytic domains facing each other and that this arrangement was required for catalytic activity [36]. A modification of one of the HDACs is all that is required to inhibit the activity of the HDAC dimer.

### Epigenetic mechanisms directed by noncoding RNAs

A widespread effect of HDAC inhibitors on microRNAs (miRNAs) levels was first reported in the breast cancer cell line SKBr3. Following a short exposure of these cells to a proapoptotic dose of the HDAC inhibitor LAQ824 (also known as dacinostat), significant changes in the levels of 40% of the miRNA population were detected. The majority of miRNAs were downregulated, but some were upregulated [88]. miRNAs are short, noncoding RNAs (ncRNAs) of about 23 nucleotides that regulate gene expression at the posttranscriptional level by binding to the 3'-UTRs of target mRNAs, leading to their degradation or translation repression. Although the biogenesis of miRNAs is well-understood, little is known of the regulation of miRNA expression, but there is increasing evidence that miRNA expression is widely misregulated in tumors, with tumor suppressor miRNAs targeting growth-inducing genes being downregulated and oncogenic miRNAs targeting growth-inhibiting genes being upregulated [89,90].

Similarly, misregulation of miRNA expression is characteristic of metastasis [91]. miRNAs are initially transcribed by RNA polymerase II into long-coding or noncoding intragenic or intergenic RNAs. They can overlap RNA transcripts in the same, opposite or both directions. Noncoding intragenic RNAs transcribed in the same direction as the coding RNA can be transcribed from the same promoter as the host RNA or can have their own promoter embedded in an intron. Promoters used by other ncRNAs are mostly unknown [90]. Some promoters are associated with CpG islands, which can become hypo- or hypermethylated during tumorigenesis, resulting in transcriptional activation or silencing, respectively. Researchers in a number of studies have demonstrated the reversal of specific miRNA transcriptional silencing following treatment with DNA methyltransferase (DNMT) and/or HDAC inhibitors, indicating that these anticancer agents can indirectly induce posttranscriptional repression of target genes [89,90,92-94]. Investigators in one study showed that transcriptional silencing of miR-22 in acute lymphoblastic leukemia cells was independent of DNA methylation of the CpG island within the promoter region, but entailed K27 trimethylation of associated H3 histone. miR-22 transcriptional silencing could be reversed by TSA treatment [95]. In acute myeloid leukemia (AML), miR-223 was shown to be a direct transcriptional target of the AML1/ETO fusion oncoprotein resulting from the t(8;21) translocation. By recruiting HDAC1, DNMT and MeCP2 activities, AML1/ETO induces heterochromatic silencing of miR-223 [96].

Alternatively, it has been shown that miRNAs can regulate genes encoding epigenetic regulators such as DNMT3a and DNMT3b, Polycomb-associated K-methyltransferase EZH2 (also known as KMT6), HDAC1 and HDAC4 [89,90]. It was determined that the *HDAC1 *gene, a direct target of miR-449a, was upregulated in prostate cancer cells and tissues as a consequence of miR-449a downregulation [97]. Similarly, *HDAC4 *targeted by miR-1-1 was upregulated in human hepatocellular carcinoma cells and primary hepatocellular carcinoma [98]. During mouse development, HDAC4 plays a crucial role in the regulation of skeletogenesis and myogenesis, and its expression has been shown to be controlled at the posttranscriptional level by miR-140 and miR-1, respectively [99,100].

More recently, the existence of another class of miRNAs mediating transcriptional gene silencing (TGS) was demonstrated [101,102]. Contrary to miRNA-mediated posttranscriptional silencing, which is transient and dependent on the sustained presence of the effector miRNA, TGS targets promoter regions and triggers heterochromatin formation by inducing DNA and histone methylation (H3K27me3 and H3K9me2), which leads to long-term silencing. This process can be inhibited by TSA, indicating a role for HDACs. The mechanism implicated in the chromatin reorganization in the promoter region of the target gene is not fully understood, but it is known to involve RNA-RNA pairing between the small RNA and the nascent RNA transcript and, among other factors, EZH2, DNMT3A and HDAC1 [103,104]. Moreover, in miRNA-induced TGS of HIV-1, heterochromatin formation was initiated at the nucleation center, as directed by the miRNA, and was further extended both upstream and downstream to include adjacent genes. It was directly shown that HDAC1 was involved in this process [105]. As a side note, miRNAs are also able to activate transcription, notably of the gene *p21*, for example, perhaps by targeting and repressing a repressor miRNA [103,104].

Long, noncoding RNAs (lncRNAs) have been known to recruit Polycomb proteins and associated HDACs to initiate and maintain heterochromatin formation in the silencing of developmentally important genes, such as in X-chromosome inactivation and parental imprinting [104,106]. It has been proposed that long, single-stranded ncRNAs are integral components of chromatin, which may stabilize binding of nonhistone proteins to chromatin, such as the heterochromatin protein 1 (HP1), and/or may play a role similar to that of H1, a linker histone contributing to the formation of higher-order chromatin structures. It is possible that RNA could facilitate the folding of the chromatin fiber and the formation of loops involving long-distance interactions [107].

The role of ncRNAs in the regulation of gene expression is beyond the scope of this review, but it has become evident that the majority of protein-coding genes are regulated by antisense RNAs and, moreover, that a large part of the genomes of humans and other complex organisms consist of non-protein-coding DNA sequences, which are transcribed. It has been suggested that these noncoding regions, previously termed "junk DNA," supply a vast array of ncRNAs which control the different epigenomes supporting development and generated throughout life in response to diet and environment. There is increasing evidence that misregulation by ncRNAs is also responsible for cancers and other diseases. Furthermore, it has been proposed that ncRNAs "provide the regulatory power and plasticity required to program our ontogeny and cognition" [106, p1610].

### Role of phosphorylation in targeting of class I histone deacetylases

**Figure 2:**
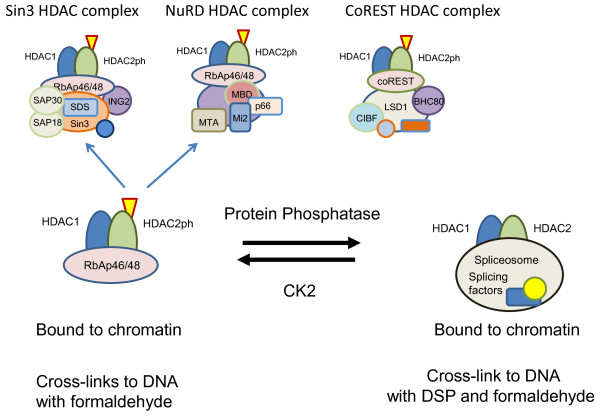
**Model for the regulation of HDAC1-HDAC2 complex formation by phosphorylation**. When phosphorylated by CK2, HDAC2 binds to the core components of Sin3, NuRD and CoREST complexes as homodimer or heterodimer with HDAC1. In the low or unphosphorylated states, HDAC1 and HDAC2 bind to proteins such as the serine/arginine (SR)-rich proteins and RNA-binding protein Hu antigen R (HuR/ELAVL1) which interact with the spliceosome.

Phosphorylation of HDAC1 (S393, S421, S423), HDAC2 (S394, S422, S424) and HDAC3 (S424) stimulates enzyme activity [108,109]. *In vitro *HDAC2 is phosphorylated by casein kinase (CK2), whereas HDAC1 can be phosphorylated by CK2, cAMP-dependent protein kinase and protein kinase G [25]. This difference constitutes more evidence that, although they share a high degree of homology, HDAC1 and HDAC2 have distinct and separately regulated functions. The regulation of HDAC1 and HDAC2 activities by PTMs has recently been reviewed [109]. Phosphorylation of HDAC1 and HDAC2 is also required for their incorporation into the Sin3, NuRD and CoREST corepressor complexes [25,41,110]. Recruitment of HDAC2 to regulatory regions of target genes by transcription factors also depends on its highly phosphorylated state [41]. On the other hand, the nonphosphorylated or monophosphorylated HDAC2 is associated with coding regions of transcribed genes [41] (Figure 2). Although unmodified and monophosphorylated HDAC2 are more abundant than highly phosphorylated HDAC2, it is the highly phosphorylated form that is preferentially cross-linked to chromatin with formaldehyde or cisplatin [111]. Thus, under conditions typically used in ChIP, highly phosphorylated HDAC2, but not unmodified or monophosphorylated HDAC2, is preferentially cross-linked to nuclear DNA *in situ *with formaldehyde. Through the use of a dual cross-linking ChIP assay, however, all isoforms of HDAC1 and HDAC2 could be mapped along regulatory and coding regions of transcribed genes, with the monophosphorylated or unmodified HDAC2 being associated with the coding region [41].

It should be noted that HDAC phosphorylation is dynamic and dependent on the balance of opposing activities of involved kinases and phosphatases. Treatment of cultured cells with the protein phosphatase inhibitor okadaic acid resulted in HDAC1 and HDAC2 hyperphosphorylation concomitant with the dissociation of HDAC1 and HDAC2, as well as the dissociation of HDAC1 from mSin3A or YY1. On the other hand, the HDAC1 and HDAC2 interactions with RbAp46 or RbAp48 were not disrupted [112]. In view of the above-described results [25,41,110], however, it appears that the observed dissociation of the HDAC-corepressor complexes subsequent to okadaic acid treatment are due not to the hyperphosphorylation of HDAC1 and HDAC2, but rather to the hyperphosphorylation of other unidentified factors.

A role of dynamic phosphorylation was also demonstrated for HDAC3. HDAC3 activity not only depends on its binding to the NCoR/SMRT complex but also is increased by CK2-mediated phosphorylation on S424. Conversely, HDAC3 interaction with protein phosphatase 4 results in decreased activity [113]. Moreover, it was shown that the phosphorylation state of HDAC3 had no effect on its association with NCoR or its subcellular localization [113]. In a recent study in which human HCT116 colon cancer cells were treated with sulforaphane (SFN), a cancer chemoprotective agent abundant in broccoli, a link between CK2-mediated phosphorylation of HDAC3 and SMRT and the disruption of the nuclear HDAC3-SMRT corepressor complex, followed by the export of HDAC3 into the cytoplasm, was suggested [114]. The proposed model is that 14-3-3 and Pin1 compete with each other for binding of cytoplasmic phosphorylated HDAC3, with binding to 14-3-3 eventually resulting in the reentry of HDAC3 into the nucleus and binding to Pin1 directing HDAC3 degradation. Thus extended exposure to SFN would lead to HDAC3 degradation [114]. Again, to reconcile these results with previous ones [113], it must be assumed that HDAC3 dissociation from SMRT and export to the cytoplasm are due to events other than CK2-mediated HDAC3 phosphorylation.

In the specific context of oxidative and/or nitrative stress, such as cigarette smoke, in chronic obstructive pulmonary disease (COPD), HDAC2 phosphorylation or nitration was linked to its ubiquitination and degradation by proteasome [115-117]. Corticosteroids are used to reduce inflammation in the airways of patients with COPD. The mode of action of corticosteroids includes the recruitment of HDAC2 and silencing of proinflammatory genes. However, resistance to the anti-inflammatory actions of corticosteroids occurs under oxidative and/or nitrative stress. Among a number of mechanisms contributing to this corticosteroid insensitivity, phosphoinositide 3-kinase-δ- and CK2-mediated HDAC2 phosphorylation were implicated [115,116,118]. Of note, administration of HDAC inhibitors affecting class I HDACs should not be given to COPD patients in the treatment of other diseases.

### Role of class I histone deacetylases associated with coding regions

Several studies have addressed the role and mode of recruitment of class I HDACs to the coding region of active genes. In *Saccharomyces cerevisiae*, the Rpd3S HDAC complex is recruited by the chromodomain of its EAF3 subunit and the plant homeobox domain (PHD) of its RCO1 subunit to the H3K36me mark to deacetylate nucleosomes behind the elongating RNA polymerase II, thus preventing cryptic initiation of transcription within the coding region [119-121]. In mammals, a novel complex composed of SIN3B, HDAC1, the EAF3 ortholog MRG15 and the PHD finger-containing PF1 (a homolog of yeast RCO1) was recently identified. This complex is associated with discrete loci of constitutively active genes and is believed to regulate RNA polymerase II progression within transcribed regions [122].

A role of HDAC1 in alternative pre-mRNA splicing has recently been reported in HeLa cells [123]. Alternative splicing of pre-mRNA gives rise to mature mRNA isoforms coding for functionally different proteins. This alternative splicing plays essential roles in differentiation and development as well as in diseases. There has been increasing evidence that transcription and splicing are coupled, and recent studies have revealed the contribution of chromatin structure and histone modifications to the selection of splice sites [124,125]. One of these studies addressed the role of HDACs in the regulation of alternative splicing [123]. It was shown that following treatment of HeLa cells with the HDAC inhibitor sodium butyrate, about 4% of the genes exhibited altered splicing. Further characterization of one of these genes, fibronectin (*FN1*), indicated that HDAC inhibition resulted in alternative exon skipping and was associated with increased histone H4 acetylation, increased RNA polymerase II processivity and reduced cotranscriptional association of the splicing regulator SRp40 at the target exon. Moreover, knockdown studies demonstrated that HDAC1, but not HDAC2, activity was required for the alternative splicing [123]. Although that study provided mechanistic insight into the role of HDAC1 in alternative splicing, the question of how HDAC1 is targeted to a particular intron-exon junction remains. It is possible that a newly identified class of small ncRNAs, the splice site RNAs, whose 3' ends map precisely to the splice donor site of internal exons in animals, serve as markers [126]. It is noteworthy that the genes whose splicing was affected by HDAC inhibition were all genes involved in cell fate and differentiation [123]. One of these genes encodes the tau protein, which is highly expressed in the central nervous system. It turned out that the expression of a splice variant of the tau protein upregulated in some neurodegenerative diseases was reduced following treatment with sodium butyrate. This indicates that some of the therapeutic benefits of HDAC inhibitors may be due to the modulation of alternative splicing. Furthermore, that study [123] represents another example of the different functions of HDAC1 and HDAC2. A recent study demonstrated that Hu proteins (for example, HuR/ELAVL1), which are splicing regulators, bind to HDAC2 and inhibit its enzyme activity. The Hu proteins are recruited to transcribed genes by an interaction with RNA polymerase II and transferred to pre-mRNA at Hu target sites. Inhibition of HDAC2 activity by the Hu proteins results in localized increase in histone acetylation at specific exons [127], increasing the transcriptional elongation rate.

Questions remain regarding the role of HDAC2 or the presence of HDAC3 on coding regions. A genomewide mapping study in primary human CD4^+ ^T cells showed that HDAC1, HDAC2, HDAC3 and HDAC6 were enriched in active genes. Moreover, HDAC1 and HDAC3 were present mostly in promoter regions, whereas HDAC2 and HDAC6 were localized to both promoter and coding regions of active genes [40]. However, these results conflict with those of studies in which other cell types were studied. In MCF-7 cells, HDAC1 and HDAC2 were both associated with regulatory and coding regions [38,41]. Moreover, bufexamac, a class IIb-specific HDAC inhibitor, did not affect the acetylation levels of histones in HeLa cells, suggesting that histones are not substrates of HDAC6 [53]. Nonetheless, the study showed that dynamic acetylation was associated with active chromatin characterized by the H3K4 methylation mark [40]. This observation was in agreement with results obtained in mouse fibroblast cells [83,128]. A recent study in *S. cerevisiae *reported that dynamic acetylation was required for the recruitment of splicing factors during cotranscriptional spliceosome assembly [129].

## Conclusion

One major issue with HDAC inhibitors often referred to in the literature is their lack of specificity, in particular their lack of isoform selectivity [130]. Another issue is the lack of known targets. There is still much to learn about the many ways that HDAC inhibitors affect gene expression. Indeed, we have just started to comprehend the breadth of their actions in changing gene expression in normal and abnormal states. New tools and methods are continually being developed, however, leading to discoveries that challenge our paradigms. For example, the specificity of HDAC inhibitors might be directed not toward HDAC isoforms but toward HDAC complexes. This finding underlines the importance of resolving HDAC interactions with other proteins and identifying genomic targets of HDAC complexes in relevant tissues in normal and disease states.

Classes I and II HDACs often exist as dimers. For example, class I HDACs form heterodimers with class II HDACs (for example, HDAC3-HDAC4). Furthermore, class I HDAC1 and HDAC2 form either homodimers or heterodimers. Multiprotein complexes with either an HDAC1-HDAC2 heterodimer versus an HDAC1 or HDAC2 homodimer may have different properties and substrate preferences. Discovery of the mechanism regulating HDAC1-HDAC 2 homo- versus heterodimer formation in cells will be important to understanding the biology of these enzymes.

There is still much to be learned about the mechanistic linkages between class I HDACs, transcription and RNA splicing. Whether other splicing regulators, such as Hu proteins, regulate HDAC activity will be an important question to address. The profound impact of HDAC inhibitors on the alternate splicing of RNAs and miRNAs requires further investigation to understand the full impact of the inhibitors on the cellular spectrum of RNAs and proteins. Recently developed mass spectrometry approaches are beginning to sort out the effects of miRNAs on protein profiles. Such approaches are required to understand the effect of HDAC inhibitor-altered miRNA profiles on the proteome [131].

## Abbreviations

ChIP: chromatin immunoprecipitation; CK2: casein kinase; DNMT: DNA methyltransferase; HDAC: histone deacetylase; KAT: lysine acetyltransferase; KDM1/LSD1: lysine-specific demethylase 1; MAPK: mitogen-activated protein kinase; MBD: methyl-CpG-binding domain-containing protein; MEF2: myocyte enhancer factor 2; miRNA: microRNA; NCoR or NCOR1: nuclear receptor corepressor; ncRNA: noncoding RNA; NF-κB: nuclear factor κB; NuRD: nucleosome-remodeling HDAC; PTM: post-translational modification; RbAp: retinoblastoma-associated protein; SMRT or NCOR2: silencing mediator of retinoic acid and thyroid hormone receptor; TSA: trichostatin A; UTR: untranslated region.

## Competing interests

The authors declare that they have no competing interests.

## Authors' contributions

All authors contributed to the content. All authors read and approved the final manuscript.
